# Diagnostic Assessment of Nuclear Cataracts Using a Smartphone-Attachable Slit-Lamp Device: A Cross-Sectional Study in Vietnam

**DOI:** 10.7759/cureus.73783

**Published:** 2024-11-15

**Authors:** Nguyet M Duong, Nhu Q Nguyen Vu, Hai T Le

**Affiliations:** 1 Pediatrics Department, Ho Chi Minh City Eye Hospital, Ho Chi Minh City, VNM; 2 Ophthalmology Department, IVISION Eye Center, Ho Chi Minh City, VNM; 3 Optometry Department, IVISION Eye Center, Ho Chi Minh City, VNM; 4 Optometry Department, Pham Ngoc Thach University of Medicine, Ho Chi Minh City, VNM

**Keywords:** cataract, diagnostic device, portable, recordable, smart eye camera, telemedicine

## Abstract

Purpose

The purpose of this study was to compare the accuracy and agreement in nuclear cataract opacity classification between the Smart Eye Camera (SEC) device, and the slit-lamp.

Methods

A cross-sectional study was conducted with a convenient sampling of 221 eyes from 139 patients with phakic eyes, visiting IVISION Eye Center, Ho Chi Minh City, Vietnam, from November 1, 2023, to April 30, 2024. Two grading systems, Lucio-Buratto (Buratto) and World Health Organization (WHO), were used to compare the effectiveness of the two devices, with statistical analysis using Spearman's correlation coefficient and Cohen’s kappa to evaluate the agreement level.

Results

Results showed no significant difference in Buratto and WHO grading between the two devices (p > 0.05), indicating consistency between the two measurement methods. Spearman’s correlation coefficient demonstrated a strong correlation between the results from both devices, with r = 0.797 for Buratto and r = 0.579 for WHO (p < 0.001). The reliability was confirmed by high weighted-kappa values (k = 0.774 for Buratto and k = 0.539 for WHO).

Conclusion

The SEC’s comparable effectiveness to the slit-lamp supports its potential utility in blindness-prevention screening efforts.

## Introduction

Cataracts are the leading cause of blindness worldwide. According to the World Health Organization (WHO) statistics, from 1990 to 2020, cataracts were responsible for about one-third of vision impairment cases in Southeast Asia, affecting approximately 36 million people and accounting for the highest proportion of such cases (36-55%) [1]. Although cataracts are treatable when detected on time, early diagnosis requires specialized equipment and skilled healthcare workers. This is often not feasible in many regions, particularly in rural areas.

Traditionally, diagnosing cataracts involves using a slit-lamp device - a medical tool that enables clinicians to observe the detailed structure of the eye. Despite being the standard method [2], the slit-lamp device has several drawbacks. Its bulky design and complex structure require it to be fixed on a tabletop, making it impractical for examining bedridden patients and small children who cannot sit in the proper position. In addition, its high cost limits its availability in remote healthcare facilities.

The lack of specialists makes teleophthalmology a crucial tool for improving the quality and accessibility of eye care, especially during the COVID-19 pandemic [3]. Teleophthalmology enables large-scale screenings and early detection of conditions like diabetic retinopathy [4], glaucoma [5], and age-related macular degeneration in remote areas [3,6,7]. However, the devices for capturing images are, again, extremely costly. Smartphone-attachable devices mainly focus on the posterior segment [8] and the cornea [9], with diffuse lighting, making it hard to detect abnormalities in the structure of the middle - the lens.

The Smart Eye Camera (SEC), introduced in 2019, is a portable device attached to a mobile phone’s camera with a slit-light converter, thereby recording videos and aiding in cataract diagnosis. This device was evaluated for nuclear cataract (NUC) opacity grading in Japan in 2020 on 128 eyes, showing high agreement with the slit-lamp [10]. Therefore, this study aims to assess the effectiveness of the SEC compared to the conventional slit-lamp device in grading NUC opacity in the Vietnamese population.

## Materials and methods

Study population

This cross-sectional analytical study was conducted from November 1, 2023, to April 30, 2024, at IVISION Eye Center, Ho Chi Minh City, Vietnam. The study included all patients who met the inclusion criteria and visited the IVISION Eye Center. Convenient sampling was used due to the lack of previous studies in Vietnam. The inclusion criteria were: (1) age 18 and above; (2) natural pupil dilation ≥6 mm or the ability to dilate with medication (0.5% tropicamide and 0.5% phenylephrine); (3) agreement to participate in the study, with a signed consent form. Patients meeting one or more of the following exclusion criteria were excluded: (1) both pseudophakic or aphakic eyes; (2) severe corneal opacifications affecting the evaluation results. A total of 221 eyes from 139 patients were enrolled.

This study conforms to the Declaration of Helsinki. The protocol was approved by the Ethical Review Board of Pham Ngoc Thach University of Medicine. Patient data was encoded to ensure confidentiality. Informed consent was obtained from all participants, after an explanation of the nature and possible consequences of the study.

Examination and diagnostic equipment

**Figure 1:**
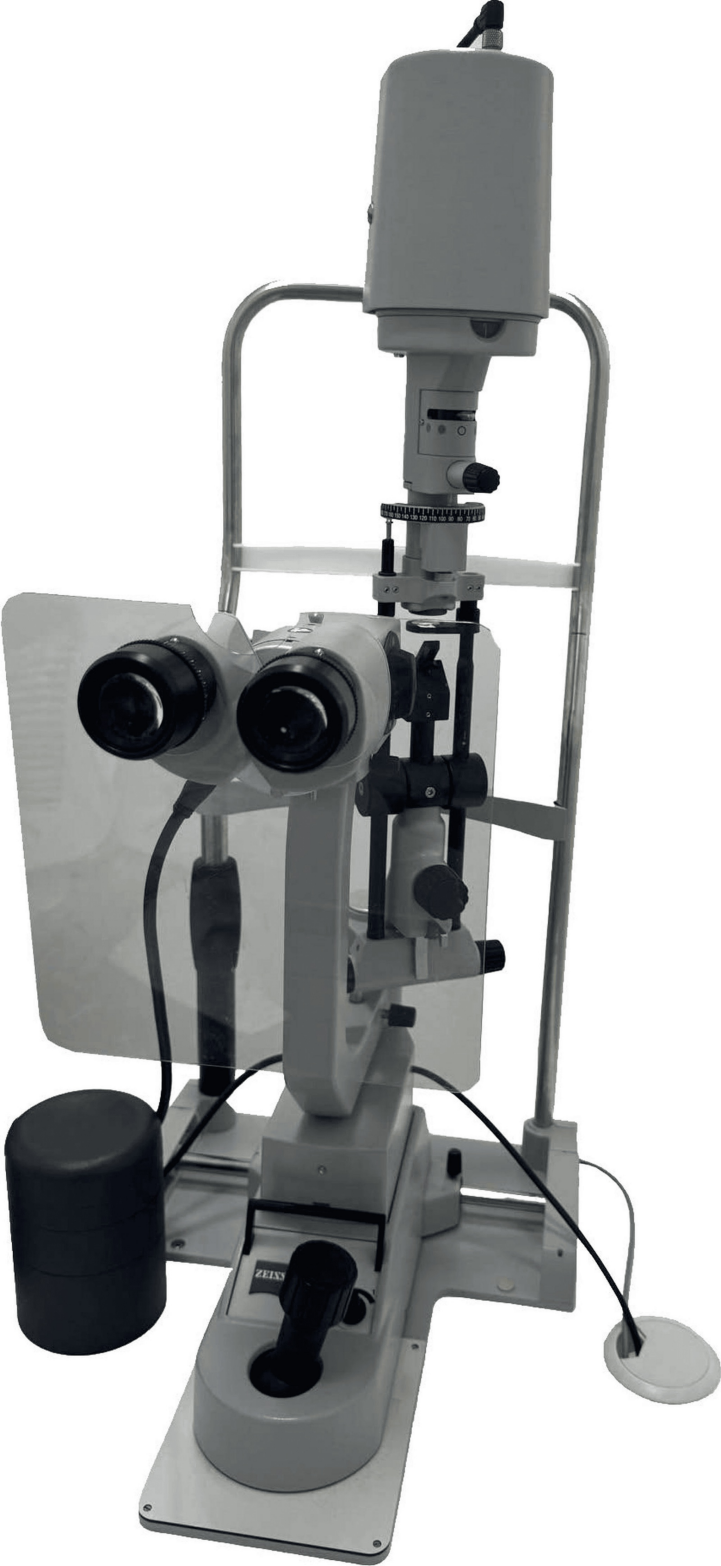
Slit-lamp device (SL-220 Carl Zeiss)

**Figure 2:**
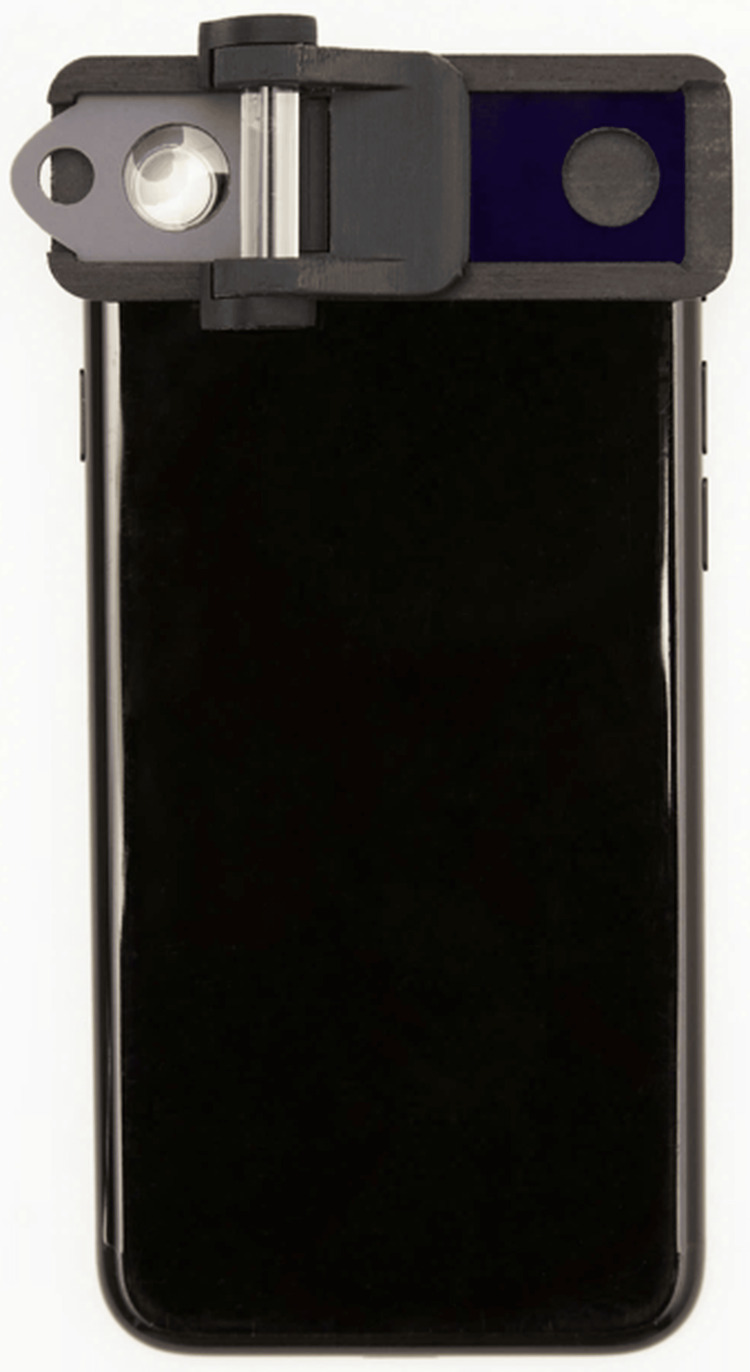
SEC device SEC attached to a smartphone (iPhone SE) SEC, Smart Eye Camera

The slit-lamp (SL-220 Carl Zeiss; Carl Zeiss Meditec AG, Jena, Germany) (Figure 1) is used for diagnosing and grading NUC opacity by traditional methods. In comparison, the SEC (Figure 2) is a device attached to a smartphone (iPhone SE; Apple Inc., Cupertino, CA, USA). This device, which has been issued a medical device code in Japan (13B2X10198030101) and registered as a Class-1 medical device in Vietnam, features a cylindrical lens mounted in front of the phone's light source, with a fixed 1-mm slit width at a 40-degree angle. Additionally, a convex macro lens is placed directly in front of the phone camera to adjust the focus on the anterior segment of the eye. The video has a resolution of 1080p and 30 frames per second.

Study protocol

**Figure 3:**
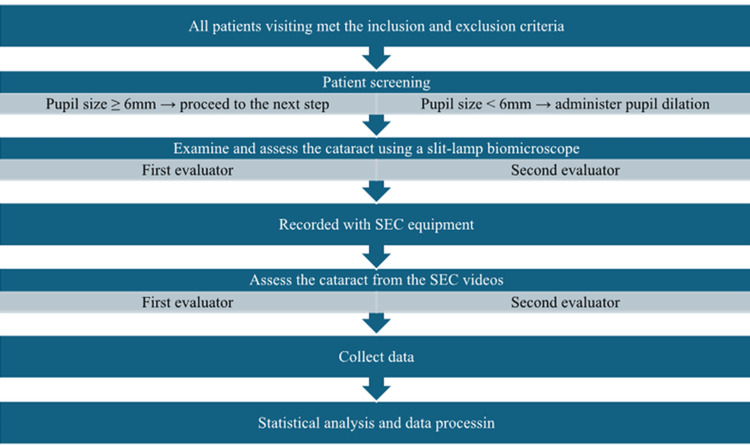
Process flowchart SEC, Smart Eye Camera

Uncorrected visual acuity was measured using the decimal chart. Pre-dilation screening was conducted, and dilating eye drops were administered if the pupil was less than 6 mm. Two ophthalmologists independently examined and assessed the degree of NUCs using a slit-lamp, recording their findings on evaluation forms. An optometrist or a nurse recorded videos of each patient’s eye using the SEC. Two other optometrists independently assessed the degree of NUCs from the videos. Both were blinded to patient information and initial assessments, as well as received prior training to ensure consistency in grading. Finally, the data were collected, processed, and statistically analyzed (Figure 3).

Capture technique

The equipment was positioned 4 cm from the patient's eye at eye level. One hand was placed on the patient’s forehead to stabilize the equipment. The patient was instructed to fixate on a target 3 m away. Video capture began when the iris occupied approximately 80% of the screen width, with the slit positioned in the center of the screen. Patients were encouraged to keep their eyes open wide, and, if necessary, the upper lid was gently held to assist. The video quality was reviewed and retaken if it did not meet the required standard.

Statistical analysis

**Figure 4:**
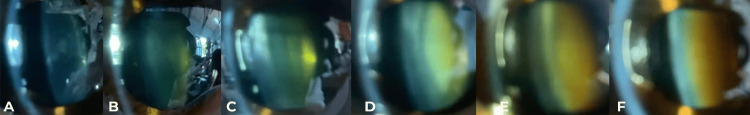
Images captured by the SEC device using the Buratto grading system (A) No nuclear opacity - Grade 0, (B) Grade 1, (C) Grade 2, (D) Grade 3, (E) Grade 4, (F) Grade 5 SEC, Smart Eye Camera

**Figure 5:**
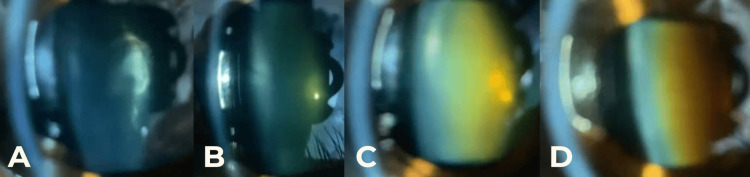
Images captured by the SEC device using the WHO grading system (A) NUC-0 with posterior polar cataract, (B) NUC-1, (C) NUC-2, (D) NUC-3 SEC, Smart Eye Camera; WHO, World Health Organization; NUC, Nuclear Cataract

All data were analyzed using IBM SPSS Statistics for Windows, Version 27 (Released 2020; IBM Corp., Armonk, NY, USA). NUC was graded using the Lucio-Buratto (Vietnam Ministry of Health) (Figure 4) [11] and WHO grading systems (Figure 5) [12]. Cohen’s kappa and Spearman correlation statistics were used to assess the level of agreement between the two methods, with statistical significance determined at p < 0.05.

## Results

Population characteristics

The study was conducted on 221 eyes from 139 patients, with a gender distribution of 31.65% male and 68.34% female, showing a significant gender difference (p < 0.05). The mean age of the patients was 67.54 ± 9.487 years. There was no significant difference in the number of right and left eyes studied (p = 0.840). The mean recording time per eye was 7.48 ± 2.434 seconds.

Comparison of NUC grading

Buratto Grading

Comparison between slit-lamp and SEC for Buratto grading showed no significant difference (p > 0.05) in both measurements. The mean ± SD for slit-lamp grading results for the first and second measurements were 2.60 ± 0.789 and 2.61 ± 0.794, respectively. For SEC, the mean results for the first and second measurements were 2.61 ± 0.753 and 2.59 ± 0.761, respectively. Although the mean Buratto grading values measured by the slit-lamp were slightly higher than those of SEC, this difference was not statistically significant (p > 0.05, according to the Friedman test). However, the cross-tabulation (Table 1) showed discrepancies in grading results between grade 2 and grade 3 cataracts using the two methods.

**Table 1:** Cross-tabulation of cataract nuclear opacification grading between SEC device and slit-lamp based on Buratto classification SEC, Smart Eye Camera; NUC, Nuclear Cataract

		NUC grading on SEC						Total
		Grade 0	Grade 1	Grade 2	Grade 3	Grade 4	Grade 5	
NUC grading on slit-lamp	Grade 0	3 (1.4%)	0 (0%)	0 (0%)	0 (0%)	0 (0%)	0 (0%)	3 (1.4%)
	Grade 1	0 (0%)	4 (1.8%)	5 (2.3%)	0 (0%)	0 (0%)	0 (0%)	9 (4.1%)
	Grade 2	0 (0%)	0 (0%)	60 (27.1%)	15 (6.8%)	0 (0%)	0 (0%)	75 (33.9%)
	Grade 3	0 (0%)	0 (0%)	16 (7.2%)	95 (43.0%)	1 (0.5%)	0 (0%)	112 (50.75%)
	Grade 4	0 (0%)	0 (0%)	0 (0%)	2 (0.9%)	18 (8.1%)	0 (0%)	20 (9.0%)
	Grade 5	0 (0%)	0 (0%)	0 (0%)	0 (0%)	0 (0%)	2 (0.9%)	2 (0.9%)
Total		3 (1.4%)	4 (1.8%)	81 (36.7%)	112 (50.7%)	19 (8.6%)	2 (0.9%)	221 (100.0%)

WHO Grading

The grading results of the first and second measurements were, respectively, 1.67 ± 0.665 and 1.66 ± 0.666. With SEC, the results of the first measurement were 1.65 ± 0.661, and the second measurement was 1.67 ± 0.658. Comparison of WHO grading between slit-lamp and SEC also showed no significant statistical difference (p > 0.05). However, there was a discrepancy in the number of cases graded NUC-1 and NUC-2 (Table 2).

**Table 2:** Cross-tabulation of cataract nuclear opacification grading between SEC device and slit-lamp based on WHO classification NUC, Nuclear Cataract; SEC, Smart Eye Camera; WHO, World Health Organization

		NUC grading on SEC				Total
		NUC-0	NUC-1	NUC-2	NUC-3	
NUC grading on slit-lamp	NUC-0	2 (0.9%)	3 (1.4%)	0 (0%)	0 (0%)	5 (2.3%)
	NUC-1	0 (0%)	53 (24.0%)	27 (12.2%)	0 (0%)	80 (36.2%)
	NUC-2	0 (0%)	28 (12.7%)	84 (38.0%)	5 (2.3%)	117 (52.9%)
	NUC-3	0 (0%)	1 (0.5%)	3 (1.4%)	15 (6.8%)	19 (8.6%)
Total		2 (0.9%)	85 (38.5%)	114 (51.6%)	20 (9.0%)	221 (100.0%)

Correlation and reliability between devices

Correlation

There was a strong correlation between cataract opacity grading according to Buratto and WHO, between the slit-lamp and SEC, with Spearman's correlation coefficient of r = 0.797 and r = 0.579, respectively (both p < 0.001) (Table 3). This reflects high consistency in grading cataract opacity according to Buratto. Although the correlation coefficient was lower for WHO grading, it still represented significant agreement between the evaluation methods, based on WHO standards.

**Table 3:** Correlation of the NUC grading between the SEC device and slit-lamp SEC, Smart Eye Camera; WHO, World Health Organization; NUC, Nuclear Cataract

	Correlation value	p-value
Buratto grading	0.797	p < 0.001
WHO grading	0.579	p < 0.001

Reliability (weighted kappa)

High weighted-kappa values between the slit-lamp and SEC for Buratto grading (k = 0.774, 95% CI: 0.703-0.845, p < 0.001) and WHO grading (k = 0.539, 95% CI: 0.437-0.641, p < 0.001) indicated strong agreement in Buratto grading and moderate agreement in WHO grading (Table 4).

**Table 4:** Weighted kappa value between the SEC device and slit-lamp SEC, Smart Eye Camera; WHO, World Health Organization

	Weighted kappa	Standard error	p-value	95% confidence interval (lower bound-upper bound)	
Buratto grading	0.774	0.036	p < 0.001	0.703	0.845
WHO grading	0.539	0.052	p < 0.001	0.437	0.641

The results of the sensitivity, specificity, positive predictive value, and negative predictive value of the two devices in assessing the degree of NUC opacity according to Buratto grading (Table 5) and WHO grading (Table 6) are shown in this study.

**Table 5:** Sensitivity, Specificity, PPV, and NPV of two devices in assessing NUC opacity according to Buratto grading sens, Sensitivity; spec, Specificity; PPV, Positive Predictive Value; NPV, Negative Predictive Value; CI, Confidence Interval; NUC, Nuclear Cataract

	sens (95% CI)	spec (95% CI)	PPV	NPV
Grade 0	100% (100% to 100%)	100% (100% to 100%)	100%	100%
Grade 1	44.44% (11.98% to 76.91%)	100% (100% to 100%)	100%	97.75%
Grade 2	65.93% (56.20% to 75.67%)	100% (100% to 100%)	100%	83.85%
Grade 3	85.59% (79.05% to 92.12%)	99.21% (97.68% to 100%)	98.95%	88.73%
Grade 4	90% (76.85% to 103.15%)	100% (100% to 100%)	100%	99.02%
Grade 5	100% (100% to 100%)	100% (100% to 100%)	100%	100%

**Table 6:** Sensitivity, Specificity, PPV, and NPV of two devices in assessing NUC opacity according to WHO grading sens, Sensitivity; spec, Specificity; PPV, Positive Predictive Value; NPV, Negative Predictive Value; CI, Confidence Interval; WHO, World Health Organization; NUC, Nuclear Cataract

	sens (95% CI)	spec (95% CI)	PPV	NPV
NUC-0	40.0% (11.8% to 76.9%)	100% (100% to 100%)	100%	98.6%
NUC-1	66.3% (55.4% to 75.7%)	96.5% (95% CI: 91.3% to 98.6%)	93.0%	80.1%
NUC-2	71.8% (63.0% to 79.2%)	70.3% (95% CI: 60.8% to 78.3%)	73.7%	68.3%
NUC-3	78.9% (56.7% to 91.5%)	97.5% (95% CI: 94.3% to 98.9%)	75.0%	98.0%

## Discussion

The average age in our study was 67.54 ± 9.487 years, slightly younger than the 73.95 ± 9.28 years reported by Yazu et al. [10]. This age range (65-75 years) is significant, as NUCs typically cause substantial symptoms in this demographic. A meta-analysis indicates that cataracts in individuals aged 60-79 result in severe visual impairment, especially in the 70-79 age group [13]. Approximately 75% of patients aged 60-80 suffer from significant NUCs [14], suggesting they are more likely to seek medical attention.

Our study showed a higher proportion of females (68.34%) compared to males (31.65%), contrasting with the almost equal gender ratio in Yazu et al.'s study [10]. This discrepancy highlights a potential gender-related bias in Vietnam, as historically, women have received less eye care. A global study estimated that 64% of blindness cases occur in women, especially in developing countries [15]. Despite the small sample size and limited generalizability, our findings suggest increased awareness and attention to eye care among women in urban areas, like Ho Chi Minh City.

The average imaging time per eye in our study was 7.48 ± 2.434 seconds, significantly shorter than the 30.38 ± 6.27 seconds for both eyes in the Japanese study [10]. This shorter imaging time may contribute to the lower correlation and weighted kappa values. However, the correlation values are still acceptable, given the primary purpose of the SEC, which is designed to replace the conventional slit-lamp in remote areas with limited medical resources.

Our study found no significant differences in correlation levels using the Buratto and WHO grading systems between the slit-lamp and SEC. This suggests that SEC can be an effective diagnostic tool for NUCs, providing quick imaging for mass screenings in various environments, from hospitals to remote areas with limited facilities. However, lower correlation and agreement values for the WHO grading system may result from shorter imaging times, personnel variability, participants' cooperation level, and environmental conditions like lighting. The fixed intensity and immobility of the SEC's light source, as well as differences in Kelvin temperature processing by phone cameras, could impact color accuracy, influencing grading outcomes. Color accuracy could be improved if the equipment were compatible with later models of smartphones or other phone types. Currently, the SEC can only attach to a limited range of phone models (iPhone 7, 8, SE2, and SE3), restricting its accessibility to users with different devices. Expanding compatibility to more smartphone types would be beneficial. In addition, environmental factors, especially lighting conditions, may also impact image quality. In our study, lighting settings were not assessed due to a lack of expertise and equipment. Therefore, future studies should focus on evaluating the equipment under varied lighting conditions, such as dim light, bright light, darkness, and sunlight. While SEC is a useful screening tool, it cannot replace the slit-lamp, and confirmatory examinations are recommended before making clinical decisions.

Additionally, our study explored the correlation and consistency using the Buratto grading system, which is commonly used by Vietnamese ophthalmologists. The higher correlation and agreement values with the Buratto system (Spearman r = 0.797 vs. WHO r = 0.578; weighted kappa r = 0.774 vs. WHO r = 0.539) suggest that Vietnamese clinicians are more proficient in grading NUCs using the Buratto system. We also evaluated the devices' correlation and consistency in assessing other types of cataracts (posterior subcapsular, cortical, and polar). The correlation and agreement values for posterior subcapsular cataracts (K > 0.70) and moderate-high levels for cortical and polar cataracts (K > 0.60) indicate acceptable reliability. However, due to limited sample sizes, these findings require further investigation.

A novel aspect of our study is calculating sensitivity, specificity, and other reliability indices for SEC in grading NUCs. For the Buratto grading system, specificity and predictive values were high across all levels. Sensitivity was moderate-low for grades 1 and 2, but moderate-high for grades 3 and 4. The discrepancies observed in grading accuracy for lower grades, such as grade 1 or NUC-1, suggest that certain technical limitations or user-dependent factors may impact the SEC’s performance in these cases. For example, lower-grade NUCs may be subtler and less visually distinct, making accurate assessment challenging without advanced imaging capabilities. The device, while effective as a screening tool, may lack the sensitivity to reliably capture these finer gradations, especially when compared to the slit-lamp's more precise optics. Additionally, variability in user technique, such as differences in positioning, lighting, and focus, could introduce inconsistencies, particularly when grading less severe cases, where slight variations might significantly affect the outcome. Furthermore, patient cooperation plays a crucial role in obtaining accurate results. If a patient is unable or unwilling to maintain proper positioning, fixate on the target, or keep their eye open as required, the quality of the images or videos captured can suffer, potentially leading to inaccurate grading. This issue is particularly relevant when screening in community or field settings, where patients may not be as familiar with examination protocols. However, in practice, differentiating between grades 1 and 2 is less significant, as patients often have mild symptoms manageable with non-invasive aids. Similarly, WHO grading showed low sensitivity for NUC 0-1 but moderate sensitivity for NUC 2-3. Other indices were moderately high across all grades, although the negative predictive value for NUC-2 was slightly lower (<70%), possibly due to factors like personnel, imaging time, and environmental conditions. Despite that, the difference in sensitivity between grade 0 and the other grades can still answer the question of whether cataracts are present or not. This demonstrates that the device can be effectively used for screening patients for cataracts.

Our study has several strengths compared to others. It provides an in-depth evaluation of SEC's reliability and agreement in grading NUCs. In addition, it assesses SEC's consistency in grading other cataract types, paving the way for future research. Using both Buratto and WHO grading systems, the study enhances its relevance in the local context. Additionally, it calculates reliability indices for SEC, offering a comprehensive evaluation of its diagnostic performance.

However, the study's limitations include being conducted at a single center with a limited sample size and lack of racial diversity, which affects generalizability. Future research should expand the study scale and include multiple sites, diverse populations, and various clinical conditions to enhance generalizability. The shorter imaging time may contribute to the lower correlation and weighted kappa values compared to previous studies. Regarding environmental settings, we did not specifically assess this factor due to a lack of equipment and tools. We recommend that future studies address this aspect. Nevertheless, our results under standard clinical conditions were quite acceptable. Additionally, if this camera is intended for use in remote areas, testing under minimal conditions would be advisable in future studies.

## Conclusions

In conclusion, the study demonstrated that both the slit-lamp and SEC are effective tools for grading NUCs using the Buratto and WHO grading systems. The findings suggest that mobile devices like the SEC are not only convenient but also reliable and precise, supporting their use in clinical settings. Proving the effectiveness of such technology can promote its broader acceptance in medical diagnostics and teleophthalmology, ultimately enhancing accessibility to eye care services. This advancement is particularly significant in efforts toward preventing blindness, especially in underserved or remote areas.
